# Effects of Hyperbaric Oxygen on Metabolic Capacity of the Skeletal Muscle in Type 2 Diabetic Rats with Obesity

**DOI:** 10.1100/2012/637978

**Published:** 2012-06-18

**Authors:** Naoto Fujita, Fumiko Nagatomo, Shinichiro Murakami, Hiroyo Kondo, Akihiko Ishihara, Hidemi Fujino

**Affiliations:** ^1^Department of Rehabilitation Science, Kobe University Graduate School of Health Sciences, 7-10-2 Tomogaoka, Suma-ku, Kobe 654-0142, Japan; ^2^Laboratory of Cell Biology and Life Science, Graduate School of Human and Environmental Studies, Kyoto University, Yoshida-Nihonmatsu-cho, Sakyo-ku, Kyoto 606-8501, Japan; ^3^Department of Physical Therapy, Himeji Dokkyo University, 7-2-1 Kamiohno, Himeji 670-8524, Japan; ^4^Department of Food Sciences and Nutrition, Nagoya Women's University, 3-40 Shioji-cho, Mizuho-ku, Nagoya 467-8610, Japan

## Abstract

We investigated whether hyperbaric oxygen enhances the oxidative metabolic capacity of the skeletal muscle and attenuates adipocyte hypertrophy in type 2 diabetic rats with obesity. Five-week-old male Otsuka Long-Evans Tokushima fatty (OLETF) and Long-Evans Tokushima Otsuka (LETO) rats were used as diabetic animals and nondiabetic controls, respectively, and assigned to control and hyperbaric oxygen groups. Animals in the hyperbaric oxygen group were exposed to an atmospheric pressure of 1.25 with an oxygen concentration of 36% for 3 h daily. The glucose level at 27 weeks of age was significantly higher in OLETF rats than in LETO rats, but the elevation was inhibited in OLETF rats exposed to hyperbaric oxygen. The slow-to-fast fiber transition in the skeletal muscle was observed in OLETF rats, but the shift was inhibited in OLETF rats exposed to hyperbaric oxygen. Additionally, the oxidative enzyme activity of muscle fibers was increased by hyperbaric oxygen. The adipocyte size was larger in OLETF rats than in LETO rats, but hypertrophied adipocytes were not observed in OLETF rats exposed to hyperbaric oxygen. Hyperbaric oxygen enhances glucose and lipid metabolism in the skeletal muscle, indicating that hyperbaric oxygen can prevent elevation of glucose and adipocyte hypertrophy in diabetic rats with obesity.

## 1. Introduction

Overeating and physical inactivity induce an increased energy intake and result in the enlargement of white adipocytes [1]. It is well known that hypertrophied adipocytes induce obesity and type 2 diabetes [2]. It has been suggested that hypertrophied adipocytes result in the overexpression of tumor necrosis factor-*α*, causing the insulin resistance that often accompanies obesity [3]. Additionally, hypertrophied adipocytes reduced the expression of adiponectin in obesity, which is associated with the subsequent development of type 2 diabetes [4]. A chronically high glucose level decreases insulin sensitivity in the skeletal muscle and causes insulin resistance [5]. Therefore, skeletal muscles that are affected by insulin resistance cannot adequately dispose glucose because of impaired insulin-stimulated glucose uptake in muscle fibers.

 Skeletal muscle, which is considered the principal site of glucose metabolism, plays an important role in glucose regulation. Skeletal muscle fibers are categorized into slow-twitch oxidative (type I), fast-twitch oxidative-glycolytic (type IIA), and fast-twitch glycolytic (type IIB) types based on their contractile and metabolic properties [6]. The metabolic capacity of the skeletal muscle depends on the fiber-type distribution [6]. Substantial differences in glucose metabolism are observed in the rates of glucose uptake among muscle fiber types, and the rate of glucose uptake is greater in skeletal muscle composed presumably of type I and IIA fibers than in those of type IIB fibers [7]. Additionally, the number of glucose transporters is larger in skeletal muscles composed presumably of type I and IIA fibers compared with those of type IIB fibers [8]. Type 2 diabetes leads to disruption of metabolic capacity in skeletal muscle, which is associated with an altered distribution of muscle fiber types. Several studies revealed that skeletal muscles in rats with type 2 diabetes have a lower percentage of type I and IIA fibers and a higher percentage of type IIB fiber than nondiabetic animals [9–12]. Furthermore, a decreased mitochondrial oxidative enzyme activity of muscle fibers has been observed in rats with type 2 diabetes [10, 11, 13, 14]. Therefore, both altered distribution of fiber types and decreased mitochondrial oxidative enzyme activity of fibers in the skeletal muscle could be related to insulin resistance and impaired glucose metabolism.

 We previously designed a hyperbaric chamber with an oxygen concentrator and an air compressor and confirmed that hyperbaric oxygen enhances the oxidative metabolic capacity of skeletal muscles and their fibers [15]. The hyperbaric chamber can maintain elevated atmospheric pressure and oxygen concentration; an elevation in atmospheric pressure accompanied by an increase in oxygen concentration enhances the partial pressure of oxygen and increases the blood flow and the concentration of dissolved oxygen in blood plasma. Both elevated atmospheric pressure and oxygen concentration activate mitochondrial oxidative enzyme activity in skeletal muscles and their fibers and cause a shift from fast to slow fibers [16, 17]. The efficacy of hyperbaric oxygen on type 2 diabetes has been reported; hyperbaric oxygen prevents the decrease in the percentage of type I and IIA fibers in skeletal muscle [9, 10, 12] and consequently inhibited an increase in the glucose level [18]. Although the enhanced oxidative metabolic capacity of skeletal muscles induced by hyperbaric oxygen decreases the glucose level in obese and type 2 diabetic subjects, its influence on adipocytes is yet unknown. Obesity-related insulin resistance is associated with the size of adipocytes, and the presence of hypertrophied adipocytes drives the development of insulin resistance [19]. Hypertrophied adipocytes may promote the infiltration of macrophages into adipose tissue, and the cytokines released from macrophages lead to the development of insulin resistance [20]. We hypothesized that hyperbaric oxygen enhances the oxidative metabolic capacity of skeletal muscles, preventing the adipocyte hypertrophy that is associated with insulin resistance. The purpose of the present study was to investigate whether hyperbaric oxygen attenuates adipocyte hypertrophy in type 2 diabetic rats with obesity.

## 2. Materials and Methods

### 2.1. Experimental Design

This study was approved by the Institution Animal Care and Use Committee and carried out according to the Kyoto University Animal Experimentation Regulation. All experiments were conducted in accordance with the National Institutes of Health Guidelines for the Care and Use of Laboratory Animals (National Research Council, 1996). 

Five-week-old male Otsuka Long-Evans Tokushima fatty (OLETF, *n* = 11) rats were used as type 2 diabetic animals with obesity, and age-matched male Long-Evans Tokushima Otsuka (LETO, *n* = 9) rats were used as nondiabetic control animals without obesity. All rats were randomly assigned to control hyperbaric oxygen groups. The abbreviations for the groups are as follows: LC, LETO control; LH, LETO exposed to hyperbaric oxygen; OC, OLETF control; and OH, OLETF exposed to hyperbaric oxygen. We designed a hyperbaric chamber for animal experiments that consisted of an oxygen tank with an oxygen concentrator and an air compressor [15]. The chamber was 180 cm long and 70 cm in diameter, and the hyperbaric environment was maintained automatically by a computer-assisted system. The animals in the hyperbaric oxygen group were exposed to an atmospheric pressure of 1.25 with an oxygen concentration of 36% for 3 h daily from 5 to 27 weeks of age. The optimal atmospheric pressure and oxygen concentration were based on the results of a previous study that obtained effective responses with regard to oxidative metabolic capacity in the neuromuscular system [15]. All animals were kept in a controlled environment with fixed 12 : 12 h light : dark cycles and room temperature maintained at 22 ± 2°C, excluding exposure to hyperbaric oxygen. Food and water were provided ad libitum for all groups.

### 2.2. Assessment of Blood Glucose Level

At 27 weeks of age, all animals were anesthetized deeply with an intraperitoneal injection of sodium pentobarbital (50 mg/kg) and blood samples were obtained from the inferior vena cava. The levels of glucose and glycated hemoglobin (HbA1c) were determined in the blood samples.

### 2.3. Assessment of Skeletal Muscle Fibers and Adipocytes

After blood sampling, the extensor digitorum longus muscle was removed and immediately frozen in acetone cooled by dry ice and stored at −80°C until the analysis. Serial transverse sections of 10 *μ*m thickness were cut on a cryostat (CM-1510S, Leica Microsystems, Mannheim, Germany) from the middle part of the muscle belly in the extensor digitorum longus muscle and mounted on glass slides. The sections were stained for myofibrillar adenosine triphosphatase (ATPase) to categorize the muscle fiber types as type I, IIA, or IIB on the basis of a previous study [21]. For ATPase staining, the sections were preincubated in barbital acetate buffer (pH 4.45) for 5 min at room temperature. Following wash by 0.1 M barbital buffer containing 0.18 M CaCl_2_ (pH 9.4) for 30 sec, the sections were incubated in 0.1 M barbital buffer containing 0.18 M CaCl_2_ and 4 mM ATP (pH 9.4) for 45 min at room temperature. The sections were then washed in 1% CaCl_2_ and in 2% CoCl_2_ for every 3 min and washed in 0.01 M sodium barbital. Following washing by distilled water, the sections were visualized by 1% ammonium sulfide. The sections were also stained for succinate dehydrogenase (SDH) and cytochrome *c* to determine quantitatively the level of mitochondrial oxidative capacity. For SDH staining, the sections were incubated in 0.05 M phosphate buffer (pH 7.5) containing 0.05% nitro blue tetrazolium and 0.05 M sodium succinate for 45 min at 37°C. For cytochrome *c* staining, the sections were incubated in 0.1 M acetate buffer (pH 5.5) containing 0.002% 3,3′-diaminobenzidine, 0.1% MnCl_2_, and 0.001% H_2_O_2_ for 60 min at 37°C, and then washed in 1% CuSO_4_ for 5 min. The histochemical images stained by SDH and cytochrome *c* were digitized as gray-scale images and the values of SDH and cytochrome *c* staining intensities were expressed as optical density values. A measuring field was set over the entire muscle cross-section for the determination of muscle fiber-type distribution. At least 100 randomly selected SDH and cytochrome *c* activities of each muscle fiber type were investigated. The sections were measured using the ImageJ software program (NIH, MD, USA). In addition, the epididymal white adipose tissue was removed after the extensor digitorum longus muscle sampling and immediately frozen in acetone cooled by dry ice without fixation. The adipose tissue sample was minced to small pieces and mounted on glass slides. Minced adipose samples were stained with Sudan Red to measure adipocyte diameter and size distribution. The adipocyte diameter was measured by using the ImageJ software program.

### 2.4. Statistical Analysis

The data were expressed as mean ± SEM. Significant differences between the four groups were analyzed using one-way analysis of variance followed by Tukey HSD post hoc test. Statistical significance was set at *P* < 0.05.

## 3. Results

### 3.1. Glucose Level

**Figure 1:**
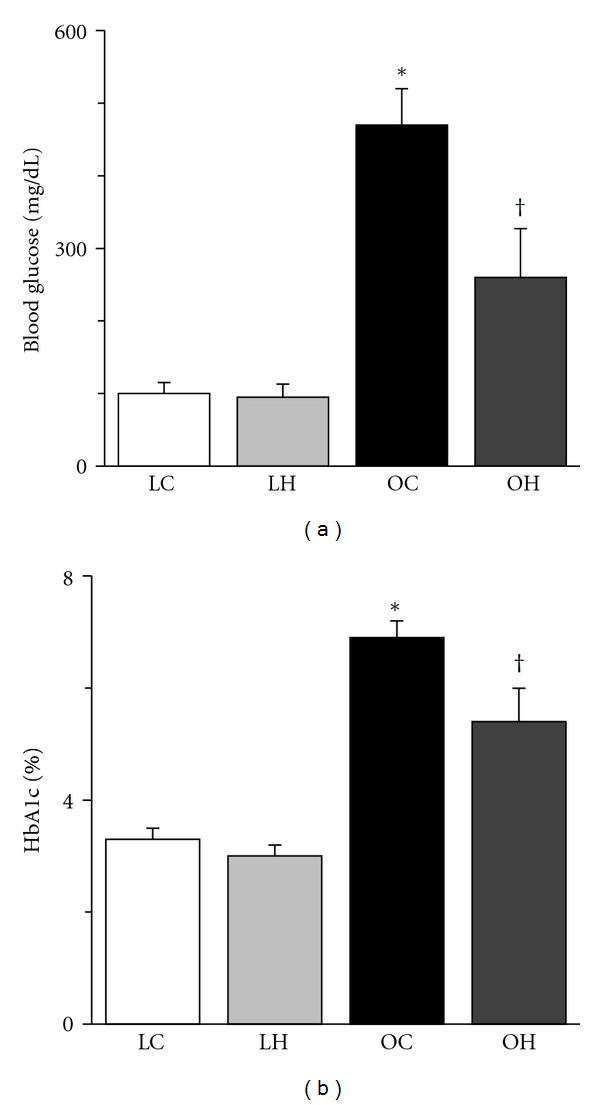
Blood glucose (a) and HbA1c (b) at the end of treatment. The values of blood glucose and HbA1c are presented as the mean ± SEM. * and ^†^ are significantly different from the LC and OC groups, respectively, at *P* < 0.05. The following abbreviations are used for Figures 1–8. LC: LETO control (non-diabetic animals); LH: LETO exposed to hyperbaric oxygen; OC: OLETF control (diabetic animals); OH: OLETF exposed to hyperbaric oxygen. *N* = 4 in the LC group, *n* = 5 in the LH group, *n* = 5 in the OC group, and *n* = 6 in the OH group.

Glucose and HbA1c levels were significantly higher in the OC group than in the LC group (Figure 1). In contrast, glucose and HbA1c levels were significantly lower in the OH group than in the OC group. No significant differences were found between the LC and LH groups.

### 3.2. Fiber-Type Distribution

**Figure 2:**
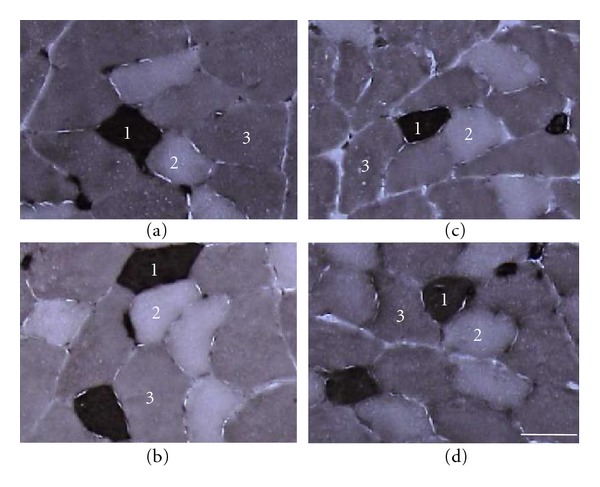
Transverse sections of the extensor digitorum longus muscle preincubated at pH 4.45 assayed for myofibrillar ATPase staining. (a) LC group; (b) LH group; (c) OC group; (d) OH group. 1: type I fiber; 2: type IIA fiber; 3: type IIB fiber. Bar = 50 *μ*m.

**Figure 3:**
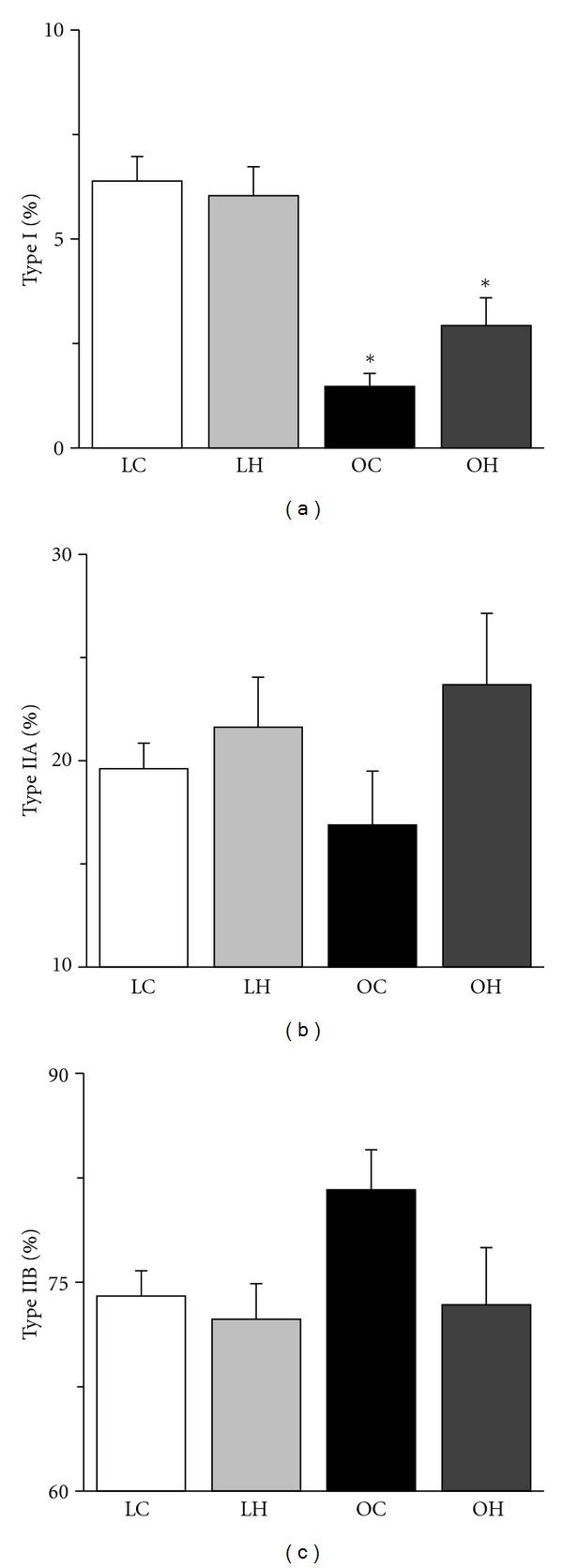
Distribution of type I (a), IIA (b), and IIB (c) fibers in the extensor digitorum longus muscle. The values of fiber-type distribution are presented as the mean ± SEM. * is significantly different from the LC and LH groups at *P* < 0.05. The measuring field was set over the entire muscle cross-section.

The ATPase staining revealed that the extensor digitorum longus muscles were composed of type I, IIA, and IIB fibers (Figure 2). The percentages of type I and IIA fibers were significantly lower in the OC group than in the LC group, and that of IIB fiber was significantly higher in the OC group (Figure 3). However, the percentages of type I and IIA fibers were significantly higher in the OH group than in the OC group, and the percentage of IIB fibers in the OH group was similar to that in the LC group.

### 3.3. Fiber Mitochondrial Oxidative Capacity

**Figure 4:**
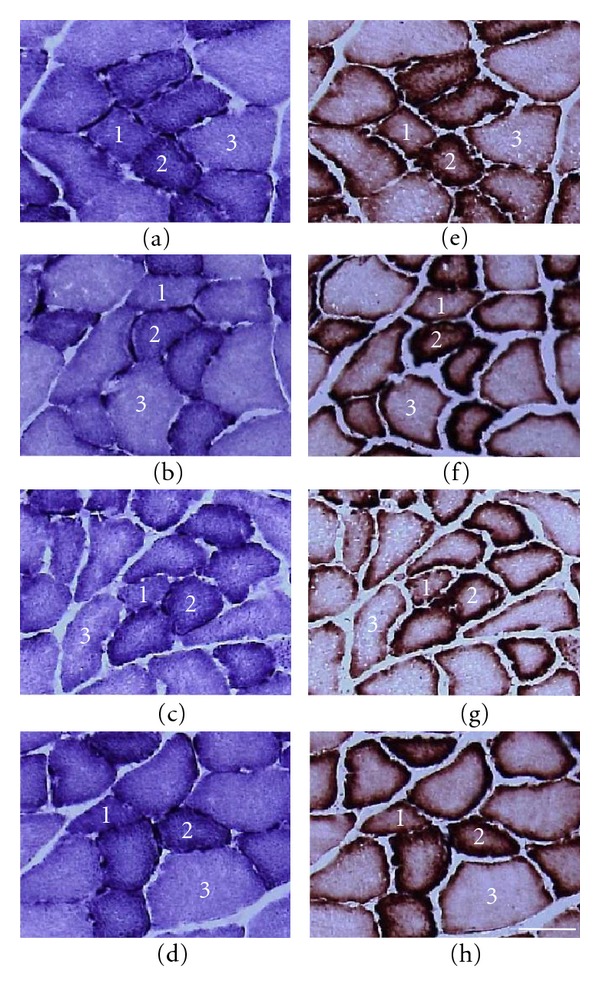
Transverse sections of the extensor digitorum longus muscle assayed for SDH ((a)–(d)) and cytochrome *c* ((e)–(h)) staining. (a) and (e) LC group; (b) and (f) LH group; (c) and (g) OC group; (d) and (h) OH group. 1: type I fiber; 2: type IIA fiber; 3: type IIB fiber. Bar = 50 *μ*m.

**Figure 5:**
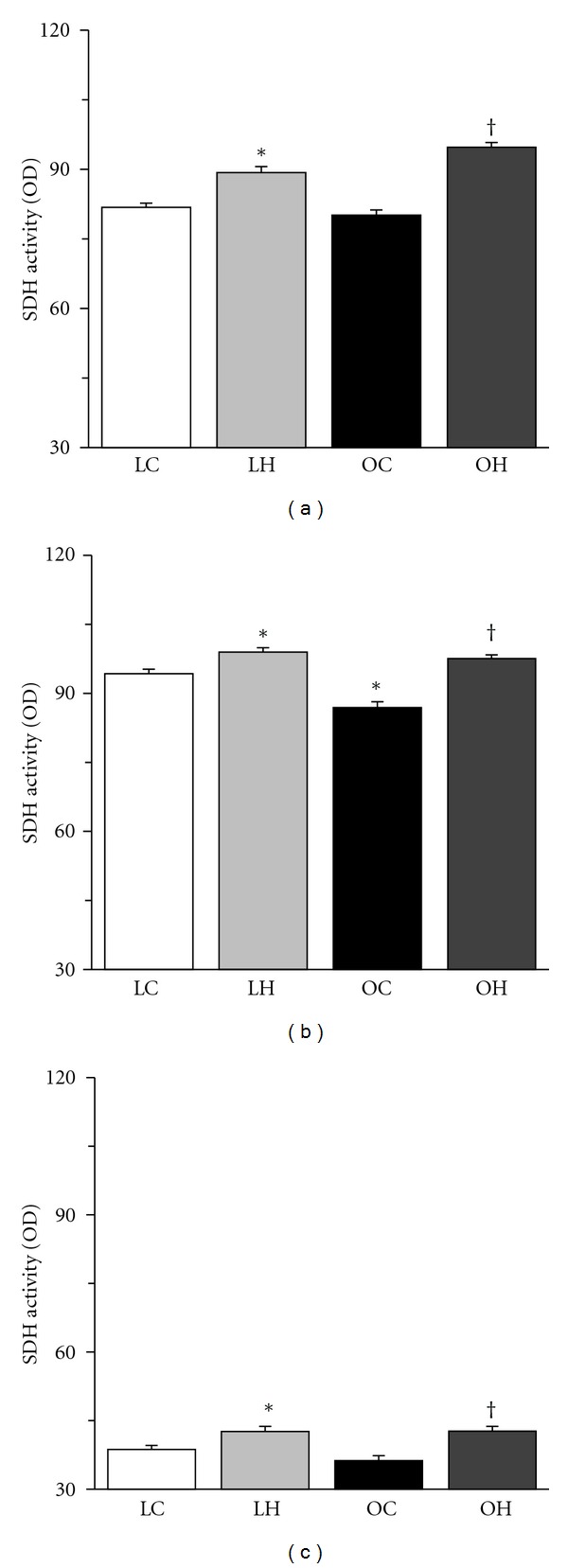
SDH activities of type I (a), IIA (b), and IIB (c) fibers in the extensor digitorum longus muscle. The values of SDH activity are presented as the mean ± SEM. * and ^†^ are significantly different from the LC and OC groups, respectively, at *P* < 0.05. In each muscle fiber type, over 100 muscle fibers were measured.

**Figure 6:**
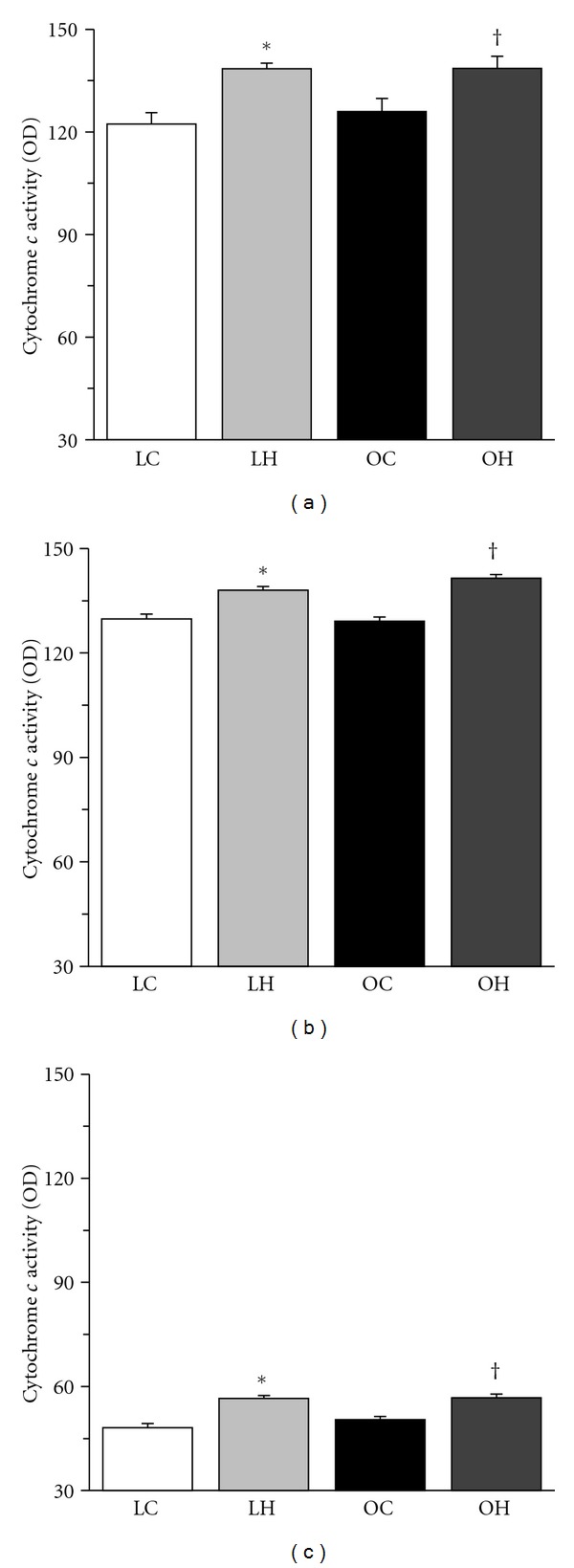
Cytochrome *c* activities of type I (a), IIA (b), and IIB (c) fibers in the extensor digitorum longus muscle. The values of cytochrome *c* activity are presented as the mean ± SEM. * and ^†^ are significantly different from the LC and OC groups, respectively, at *P* < 0.05. In each muscle fiber type, over 100 muscle fibers were measured.

For both the SDH and cytochrome *c* staining, type I and IIA fibers stained darker than type IIB fibers (Figure 4). The mitochondrial oxidative capacity of muscle fibers was increased by hyperbaric oxygen, regardless of the fiber type. The SDH and cytochrome *c* activities of the muscle fibers in the LH group were significantly higher than those in the LC group and the SDH and cytochrome *c* activities of the muscle fibers in the OH group were significantly higher than those in the OC group (Figures 5 and 6). Furthermore, type IIA fibers in the OC group had lower SDH activity than those in the LC group.

### 3.4. Adipocyte Diameter and Size Distribution

**Figure 7:**
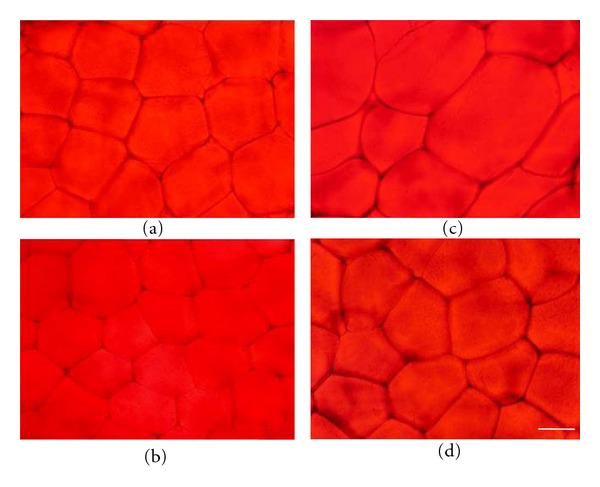
Transverse sections of the epididymal white adipose tissue were stained with Sudan red. (a) LC group; (b) LH group; (c) OC group; (d) OH group. Bar = 50 *μ*m.

**Figure 8:**
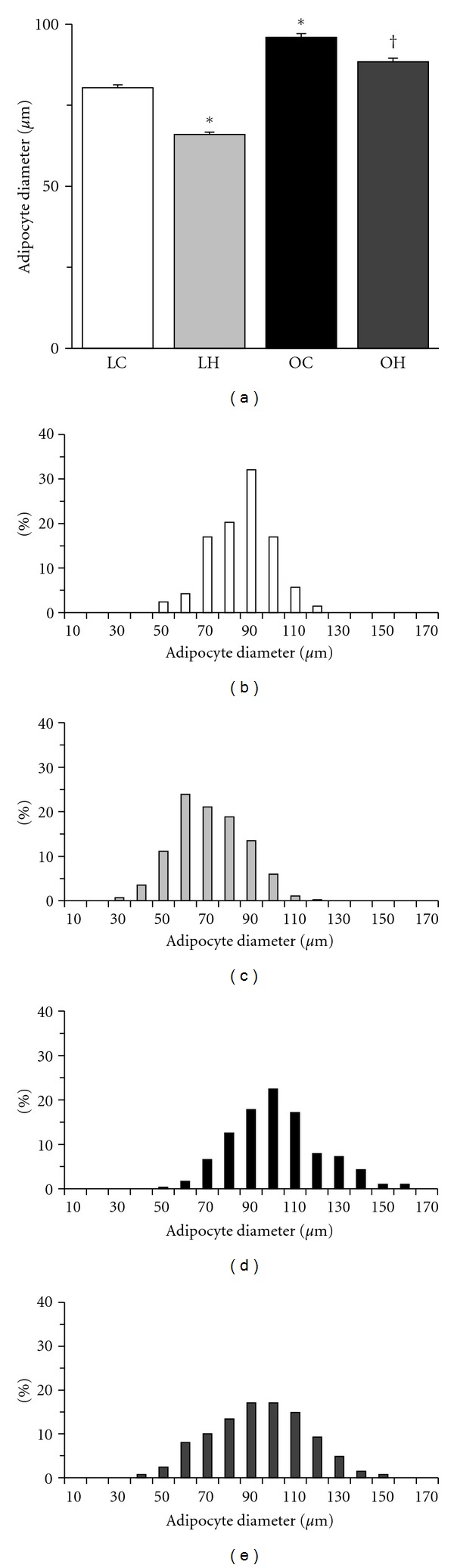
Diameter of adipocytes (a) and distribution of adipocyte size ((b) LC group; (c) LH group; (d) OC group; (e) OH group). The values of adipocyte diameter are presented as the mean ± SEM. * and ^†^ are significantly different from the LC and OC groups, respectively, at *P* < 0.05. In each adipose tissue, over 100 adipocytes were measured.

Hypertrophied adipocytes were not observed in the LC group but were abundant in the OC group (Figure 7). The diameter of adipocytes was larger in the OC group than in the LC group (Figure 8(a)), but it was significantly smaller in the OH group than in the OC group. Additionally, hypertrophied adipocytes with diameters larger than 130 *μ*m were not found in the LC group, and the percentage of these adipocytes was lower in the OH group than in the OC group (Figures 8(b)–8(e)).

## 4. Discussion

The present study revealed that hyperbaric oxygen enhances the oxidative metabolic capacity of skeletal muscles and attenuates adipocyte hypertrophy in type 2 diabetic animals with obesity. The fiber-type distribution was altered in the skeletal muscle, and elevation of glucose and adipocyte hypertrophy were observed in type 2 diabetic animals with obesity. However, hyperbaric oxygen activated mitochondrial metabolism and function in muscle fibers and prevented the transition of fibers from slow to fast in diabetic animals, thereby inhibiting the elevation of glucose and adipocyte hypertrophy.

Several studies have shown that the fiber distribution is altered in the skeletal muscle of patients and animals with type 2 diabetes [10, 12, 13, 22]. Additionally, a decreased mitochondrial oxidative enzyme activity was observed in the skeletal muscle of patients and animals with type 2 diabetes [10, 11, 13, 14, 23]. We also found that type 2 diabetic animals with obesity have a lower percentage of type I and IIA fibers and a lower mitochondrial oxidative enzyme activity in type IIA fibers compared with age-matched nondiabetic animals. In the present study, hyperbaric oxygen enhanced mitochondrial oxidative enzyme activity of all fiber types and prevented the transition of fibers from slow to fast in the skeletal muscle of rats with type 2 diabetes. The enhanced oxidative capacity of muscle fibers may be an adaptation to hyperbaric oxygen, which contributes to the increased oxidative metabolic capacity of the skeletal muscle. An increase in both the pressure and concentration of oxygen in blood plasma makes the oxygen available for diffusion into the tissues, generating an increased oxidative capacity in the skeletal oxidative muscles [10, 15]. The increased oxygen availability could have a beneficial impact on skeletal muscle oxidative metabolism. We conclude that hyperbaric oxygen prevents the altered distribution of fiber types and decreased oxidative enzyme activity of fibers in the skeletal muscles of rats with type 2 diabetes and subsequently inhibits the glucose elevation.

Type 2 diabetic rats that were not exposed to hyperbaric oxygen had a higher percentage of hypertrophied adipocytes. In contrast, the percentage decreased in type 2 diabetic rats exposed to hyperbaric oxygen. Fatty acids are transported into mitochondria and broken down to acetyl-CoA through the beta-oxidation of lipids, and the generated acetyl-CoA is used in the tricarboxylic acid (TCA) cycle in aerobic respiration to produce energy and electron carriers. The SDH is an enzyme located in mitochondria and participates in the TCA cycle followed by the electron transport system. Cytochrome *c*, which is also located in mitochondria, is a component of the electron transport system and has a role in carrying electrons. Both SDH and cytochrome *c* are related to glucose and lipid metabolism in muscle fibers. Enhanced SDH and cytochrome *c* activities in all fiber types in the skeletal muscle were observed in type 2 diabetic rats with hyperbaric oxygen in the present study. Additionally, the rats exposed to hyperbaric oxygen had a high percentage of type I and IIA fibers, which have a high fatty acid oxidation ability [24], compared with the rats not exposed to hyperbaric oxygen. These results suggest that adipocyte hypertrophy was prevented in type 2 diabetic rats with obesity because hyperbaric oxygen facilitates the turnover of glucose and lipid metabolism in the skeletal muscle.

Hyperbaric oxygen inhibited the growth-related transition of fiber types from slow to fast and the decrease in fiber oxidative enzyme activity in type 2 diabetic rats with obesity, preventing glucose elevation and adipocyte hypertrophy. These results indicate that hyperbaric oxygen enhances the glucose and lipid metabolic capacities of the skeletal muscle, which delays the development of type 2 diabetes and obesity. Increases in the glucose and lipid metabolic capacities of the skeletal muscle were also found after a long-term endurance exercise [25–27]. However, maintaining sufficient exercise training is difficult in obese patients, who often have accompanying physical dysfunctions such as osteoarthritis and myopathy. We therefore propose that hyperbaric oxygen is useful as adjunctive therapy in the treatment of type 2 diabetic patients with obesity.
